# Neuropsychological dimensions related to alterations of verbal self-monitoring neural networks in schizophrenic language: systematic review

**DOI:** 10.3389/fpsyt.2024.1356726

**Published:** 2024-03-04

**Authors:** Julián Andrés Guiral

**Affiliations:** ^1^ Humanities, EAFIT University, Medellín, Colombia; ^2^ Instituto de Neuropsicología y Lenguaje, EAFIT University, Medellín, Colombia

**Keywords:** language, verbal-self-monitoring, schizophrenic, inner speech, neural networks

## Abstract

Although schizophrenia has traditionally been interpreted as a disorder of thought, contemporary perspectives suggest that it may be more appropriate to conceptualize it as a disorder of language connectivity. The linguistic anomalies present in schizophrenia possess distinctive characteristics that, despite certain connections, are not comparable to aphasic disorders. It is proposed that these anomalies are the result of dysfunctions in verbal self-monitoring mechanisms, which may influence other neuropsychological dimensions. This study set out to examine the neuropsychological dimensions associated with alterations in the neural networks of verbal self-monitoring in schizophrenic language, based on the scientific evidence published to date. Exhaustive searches were conducted in PubMed, Web of Science, and Scopus to identify magnetic resonance studies that evaluated verbal self-monitoring mechanisms in schizophrenia. Of a total of 133 articles identified, 22 were selected for qualitative analysis. The general findings indicated alterations in frontotemporoparietal networks and in systems such as the insula, amygdala, anterior cingulate cortex, putamen, and hippocampus. Despite the heterogeneity of the data, it is concluded that language plays a fundamental role in schizophrenia and that its alterations are linked with other neuropsychological dimensions, particularly emotional and perceptual ones.

## 1 Introduction

Schizophrenia is considered a disorder of neuronal connectivity and is closely linked to language impairments. According to the DSM-5, delusions, hallucinations, and disorganized speech are the three primary symptoms of schizophrenia, and at least one of these symptoms must be present for a minimum duration of one month for an accurate diagnosis (1). While schizophrenia has traditionally been understood as a thought disorder, particularly due to the presence of these so-called positive symptoms, this categorization has become merely descriptive in contemporary discourse, as it refers to a variety of phenomena manifested in some form of verbal communication impairment (2, 3). This suggests that both delusions and hallucinations, as well as disorganized speech, have their functional correlate in various language impairments (3–5), and consequently, their neuroanatomical correlate in the neural networks that facilitate their functioning.

Language in schizophrenia, commonly known as disorganized speech or Positive Thought Disorder (PTD) (6), possesses distinct characteristics that differentiate it from other language disorders. Due to the similarities between the two, schizophrenic language has traditionally been associated with and explained through aphasic language (7), particularly due to the presence of neo logistic jargon with alliterations and assonances in both cases (8), as well as semantic aphasia, given the presence of agrammatism in both cases (9). The limitations of studying schizophrenic language from an aphasiological perspective have been highlighted by Barr et al. (10), who, from a frontal lobe dysfunction perspective, suggest that schizophrenic language is characterized by the presence of Field-Dependent Responses (FDR) and Verbal Perseverations (VP) as a result of alterations in verbal monitoring mechanisms, especially at the semantic and phonological levels. Verbal self-monitoring mechanisms, closely related to the acquisition of inner speech and consequently the regulation of behavior through speech (11, 12), provide a promising avenue for the differential study of schizophrenic language.

Verbal self-monitoring is defined as the ability to assess what is being said in relation to what was intended to be said and occurs at least on three levels: at the level of the speech command, in the realm of inner speech, and at the sensory level (13). This means that verbal self-monitoring is involved in the entire generative process of language, which encompasses sense construction, internal language, and meaning elaboration (14). It has been suggested that in schizophrenia, these mechanisms of verbal self-monitoring are impaired (15, 16). Given their relationship with the generative process of language as a whole and considering schizophrenia as a disorder of connectivity, particularly in language networks, the alteration of verbal self-monitoring mechanisms could be associated with the core symptoms of schizophrenia. In addition to these symptoms, and due to the fundamental role of language in neuropsychological development in general (11), it has been found that language impairments in schizophrenia not only affect linguistic aspects but also other cognitive, emotional, behavioral, and social domains (6).

The networks involved in cognitive self-monitoring in general have been associated with a network that includes the prefrontal and cingulate cortex, the parahippocampal region, the septal nuclei, motor regions, and the hippocampus as a hub (17, 18). According to Frith’s classic model (19) based on Positron Emission Tomography (PET) data, the verbal self-monitoring network includes the Anterior Cingulate Cortex as the main source for vocalization control, through the Broca and Wernicke areas, thereby modifying speech perception, which in turn is influenced by the thalamus. In a magnetic resonance imaging study involving various tasks related to the process of verbal self-monitoring, such as self-distorted feedback, alienated non-distorted feedback, imagining another person speaking, and monitoring externally generated speech, a significant involvement of the bilateral temporal cortex (middle and superior), auditory cortex, thalamus, cerebellum, and hippocampus was found (20).

Recent studies have investigated the relationship between language impairments in schizophrenia from various perspectives. Cavelti et al. (21) conducted a systematic review aiming to examine the neural correlates of Formal Thought Disorder (FTD) in schizophrenia and its relationship with alterations in the language network. They concluded that their hypothesis was only partially supported. Firstly, there were studies that did not find any relationship between FTD and alterations in the language network. Secondly, studies using a “whole-brain” approach revealed altered neural regions in FTD that are not part of the language network. Lastly, the high degree of heterogeneity among the studies was attributed to the multidimensionality of FTD, methodological differences, and the limited research conducted to date. On the other hand, Barber et al. (22) conducted a meta-analysis on the functional and structural neural correlates of inner speech in relation to Auditory Verbal Hallucinations (AVH) in schizophrenia. They concluded that the insula plays a fundamental role in AVH and the attribution of inner speech errors.

Despite the abundance of literature regarding the relationship between language and schizophrenia, there is no consensus on the specific nature of this relationship. The lack of consensus is partly due to the vast amount of disjointed information available. Exploring how language relates to the core symptoms of schizophrenia, the specific alterations that can occur at the level of networks and language errors, and how these alterations may impact other neuropsychological dimensions that are associated with other symptomatic manifestations of schizophrenia, are topics that have not been extensively studied. However, they can contribute to the discussion on how language is specifically involved in schizophrenia. This, in turn, can help determine whether schizophrenic language is an isolated characteristic separate from other symptoms and alterations in schizophrenia or if it forms the foundation of the disorder. Such understanding can further contribute to the neuropsychological approach to schizophrenia. Therefore, the objective of this research was to analyze the existing scientific evidence on the neuropsychological dimensions related to alterations in the neural networks of verbal self-monitoring in schizophrenic language. The aim was to test the hypothesis that alterations in the neural networks of verbal self-monitoring in schizophrenic language are associated with multiple neuropsychological dimensions.

## 2 Methods

### 2.1 Search strategy

The PRISMA guidelines were followed to conduct a systematic review, with predetermined inclusion and exclusion criteria. The selected articles were reviewed by the author and a collaborator before reaching a consensus for inclusion. Data extraction was performed by the author and reviewed by the collaborator. The Newcastle-Ottawa Scale for case-control studies was used to assess the risk of bias in the included studies.

An extensive search was conducted in the PubMed, Web of Science, and Scopus databases to identify studies on structural and functional neuroimaging using Magnetic Resonance Imaging (MRI) and its various techniques, investigating the alteration of neural correlates of verbal self-monitoring in schizophrenia and their neuropsychological effects. The literature search was conducted using the following combination of keywords: (“verbal self-monitoring” OR “speech monitoring” OR “speech self-monitoring” OR “inner speech” OR “speech error detection” OR “auditory verbal imagery”) AND (“schizophrenia” OR “schizophrenic speech” OR “schizophrenic language” OR “psychosis” OR “psychotic speech” OR “psychotic language” OR “formal thought disorder” OR “thought disorder” OR “positive thought disorder” OR “disorganized speech”) AND (“Magnetic Resonance Imaging” OR “MRI” OR “Functional Magnetic Resonance Imaging” OR “fMRI” OR “Diffusion Tensor Imaging” OR “DTI” OR “Diffusion Spectrum Imaging” OR “DSI”).

### 2.2 Selection and exclusion criteria

The selected studies met the following eligibility criteria: 1) adult patients aged between 17 and 50 years diagnosed with schizophrenia, 2) application of neuroimaging using MRI, and 3) articles written in English. All types of studies were considered, except for systematic reviews and meta-analyses. Both resting-state and task-based studies were included. There was no specific publication year criterion as all available scientific evidence up until 2023 was reviewed. Cases with comorbidities of other neuropsychiatric disorders, psychoactive substance use, or neurodegenerative diseases were excluded from the study.

### 2.3 Data extraction

The variables of interest extracted from the studies were as follows: study design, number of participants, sex, age, diagnosis, MRI category (structural/functional/effective), acquisition sequence (Spin-Echo/Inversion-Recovery/Gradient-Echo), MRI technique (spectroscopy/Diffusion Tensor Imaging/Diffusion Spectrum Imaging/Functional Magnetic Resonance Imaging), imaging scope (ROI/Whole Brain), modality (resting-state/task-based), imaging outcome measure, reported neural networks, and neuropsychological dimensions (behavioral/emotional/cognitive). Data extraction was independently performed by the author and the pair. The results were compared, and disagreements were resolved through discussion.

### 2.4 Data analysis

Meta-analysis was not conducted due to heterogeneity in definition and measurement of outcomes.

## 3 Results

**Figure 1:**
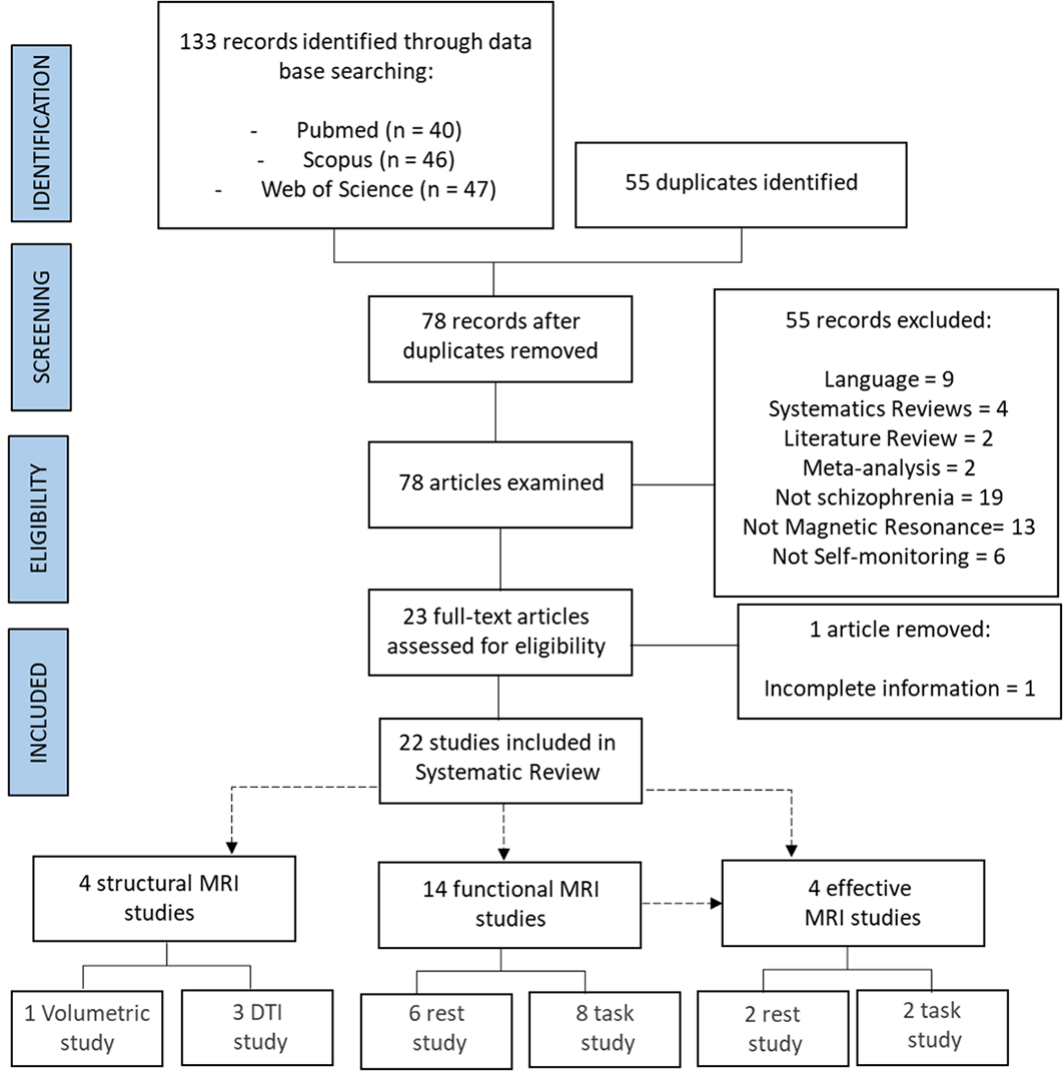
Flowchart.

With the employed search strategy, 133 articles were identified (see **Figure 1**). After excluding 55 duplicates, 78 articles were examined for eligibility. Fifty-five articles were excluded for the following reasons: articles in languages other than English (n = 9), systematic reviews (n = 4), literature reviews (n = 2), meta-analyses (n = 2), not involving patients with schizophrenia (n = 19), no use of MRI (n = 13), no reference to verbal self-monitoring or inner speech (n = 6), and incomplete information (n = 1). Finally, 22 articles met the eligibility criteria for the systematic review, which were grouped according to the type of MRI technique into structural studies (n = 4), functional studies (n = 14), and effective studies (n = 4). The analyzed studies included a total of 771 patients with schizophrenia and 630 controls. The qualitative synthesis involved extracting information on the reported neural correlates of verbal self-monitoring or inner speech and their relationship with schizophrenia, and subsequently determining the associated neuropsychological functions.

### 3.1 Structural MRI studies

#### 3.1.1 Volumetric studies

The volumetric study (**Table 1**), which utilized Local Gyrification Index (LGI) as the outcome measure, revealed lower LGI in the Broca’s Area (BA), lower LGI in the right BA and the Superior Medial Frontal Cortex (SMFC), and increased LGI in the precuneus and the Superior Parietal Cortex (SPC). Abnormalities were found in areas of the Anterior Cingulate Cortex (ACC) and the Superior Frontal Cingulate Cortex (SFCC). This study suggests that the bilateral hypogyrification of BA, ACC, and SFCC, areas closely related to language and inner speech, which in turn are associated with verbal self-monitoring, may serve as a marker for the subsequent development of Auditory Verbal Hallucinations (AVH) (24).

**Table 1:** Structural MRI studies.

Authors	Sample	Age (M/SD)	Gender (M/F)	Image Scope	Modality	Self-Monitoring Networks
Xie, S. et al. (23)	478 (AVH=113 NAVH=96 HC=269)	(26.77/5.86) (26.95/5.43) (27.04/5.82)	(56/57) (54/42) (136/133)	ROI: AF	DTI	BA, WA, AF
Kubera, K. et al. (24)	34 (AVH=10 NAVH=10 HC=14)	(36.5/9.0) (32.1/6.2) (33.7/8.6)	(6/4) (8/2) (7/7)	ROI: BA	Volumetric	BA, SPC, ACC, SFCC
de Weijer, A.D. et al. (25)	86 (AVH=44 HC=42)	(36.9/12.0) (38.4/12.6)	(25/19) (23/19)	ROI: PFC, TPJ	DTI	PFC, TPJ, BAF, CST, CC, UF
Hubl, D. et al. (26)	39 (AVH=13 NAVH=13 HC=13)	(33.3/8.5) (31.0/9.3) (32.0/8.4)	(8/5) (8/5) (8/5)	ROI: MBAF, LBAF	DTI	FTP, MBAF, LBAF, UF, SLF

AVH, Auditory Verbal Hallucinations; NAVH, No Auditory Verbal Hallucinations; HC, Healthy Controls; M/SD, mean/standard deviation; M/F, male/female; ROI, Region of Interest; DTI, Diffusion Tensor Imaging; BA, Broca’s Area; WA, Wernicke’s Area; AF, Arcuate Fasciculus; SPC, Superior Parietal Cortex; ACC, Anterior Cingulate Cortex; SFCC, Superior Frontal Cingulate Cortex; PFC, Prefrontal Cortex; TPJ, Temporoparietal Junction; BAF, Bilateral Arcuate Fasciculus; CST, Corticospinal Tract; CC, Cingulate Cortex; UF, Uncinate Fasciculus; FTP, Frontotemporal Areas; MBAF, Medial Bilateral Arcuate Fasciculus; LBAF, Lateral Bilateral Arcuate Fasciculus; UF, Uncinate Fasciculus; SLF, Superior Longitudinal Fasciculus.

#### 3.1.2 DTI studies

White Matter (WM) connectivity studies using DTI, with Fractional Anisotropy (FA) as the outcome measure (23, 25, 26), as well as measurements of the Magnetic Transfer Ratio (MTR) (25), revealed hyperconnectivity (increased FA) in the long segment of the left perisylvian network, encompassing the BA, Wernicke’s Area (WA), and the Arcuate Fasciculus (AF) (23). Furthermore, hypoconnectivity (decreased FA) was observed in the AF, Uncinate Fasciculus (UF), and Corticospinal Tract (CST), while hyperconnectivity (increased FA) was found in the Cingulate Cortex (CC).

Furthermore, hypoconnectivity (decreased FA) and degraded axons or glial cells (increased MTR) were found in the bilateral AF, which is related to the severity of positive symptoms as it hinders effective corollary discharge from the inferior frontal regions of speech to temporoparietal regions (from production to perception), leading to inadequate recognition of thoughts (25). Finally, an imbalanced directionality was found in the arcuate fasciculus, with increased directionality (increased FA) in the lateral part and decreased directionality (decreased FA) of white matter fibers in the medial part, suggesting that during inner speech, these alterations lead to abnormal coactivation in these areas related to the acoustic processing of external stimuli (26).

In conclusion, structural MRI studies suggest structural and microstructural difficulties in frontal, parietal, temporal areas, and their respective WM connections (especially in the AF), with a particular involvement of the Cingulate Cortex (CC). All of these areas and tracts are related to inner speech, but there was no reference to other related neuropsychological functions.

### 3.2 Functional MRI studies

#### 3.2.1 Resting-state studies

Alterations in Global Functional Connectivity Density (GFC) (**Table 2**) were reported in patients with AVH, showing hyperactivity in the hippocampus, bilateral insula, and most components of the default mode network (DMN), and hypoactivity in the left Temporoparietal Junction (TPJ) and components of the Frontoparietal Network (FPN) (27). Increased functional connectivity was observed between the Anterior Cingulate Cortex (ACC), insula, and language-related regions (28). Alterations in Cerebral Blood Flow (CBF) were also found in areas such as the bilateral Dorsolateral Prefrontal Cortex (DLPFC) (internal speech monitoring), Postcentral Gyri (speech production), and the right Supplementary Motor Area (SMA) (speech imagery) (29).

**Table 2:** Resting state functional MR studies.

Authors	Sample	Age (M/SD)	Gender (M/F)	Image Scope	Modality	Self-Monitoring Networks
Zhuo, C. et al. (27)	55 (AVH=20 NVAH=15 HC=20)	(25.5/2.9) (23.5/2.5) (22.0/1.6)	(7/13) (5/10) (10/10)	Whole Brain	Resting state	Hippocampus, PPC, TPJ, STG, PFC.
Chang, X. et al. (28)	54 (AVH=18 NAVH=18 HC=18)	(22.56/6.73) (22.67/3.80) (24.44/3.73)	(10/8) (9/9) (10/8)	ROI: MTG, STG, IPL, IFG, ACC, PCC, ínsula	Resting state	MTG, STG, IPL, IFG, ACC, PCC, insula.
Cui, L. et al. (29)	75 (AVH=25 NAVH=25 HC=25)	(24/7) (24/5) (26/5)	(14/11) (13/12) (12/13)	Whole Brain	Resting state	DLPFC, SMA
Clos, M. et al. (30)	98 (AVH=49 HC=49)	(37.3/11.9) (39.5/14.8)	(22/27) (19/30)	ROI: MTG, thalamus, AG, IFG	Resting State	BA, DLPFC-L, VLPFC, IFG
Vercammen, A. et al. (31)	54 (AVH=27 HC=27)	(36.67/13.13) (32.48/10.90)	(13/14) (13/14)	ROI: TPJ, IFG, ACC, amygdala, insula.	Resting state	TPJ, IFG, ACC, amygdala, insula.
Shergill, S. et al. (32)	2 (AVH=2)	(47) (26)	(2)	Whole Brain	Resting state	IFG-L, RMTG

AVH, Auditory Verbal Hallucinations; NAVH, No Auditory Verbal Hallucinations; HC, Healthy Controls; M/SD, mean/standard deviation; M/F, male/female; ROI, Region of Interest; PPC, Posterior Parietal Cortex; TPJ, Temporoparietal Junction; STG, Left Superior Temporal Gyrus; PFC, Prefrontal Cortex; MTG, Middle Temporal Gyrus; STG, Superior Temporal Gyrus; IPL, Inferior Parietal Lobule; IFG, Inferior Frontal Gyrus; ACC, Anterior Cingulate Cortex; PCC, Postcentral Gyrus; DLPFC, Dorsolateral Prefrontal Cortex; SMA, Supplementary Motor Area; AG, Angular Gyrus; BA, Broca’s Area; DLPFC-L, Left Dorsolateral Prefrontal Cortex; VLPFC, Ventrolateral Prefrontal Cortex; IPL, Inferior Parietal Lobule; IFG, Inferior Frontal Gyrus; IFG-L, Left Inferior Frontal Gyrus; RMTG, Right Middle Temporal Gyrus.

Altered coupling was reported in connections between Broca’s Area (BA), insula, SMA, and DLPFC, Ventrolateral Prefrontal Cortex (VLPFC), and Inferior Parietal Cortex (IPC) (30). Reduced connectivity was also reported between the Right Inferior Frontal Area and temporal speech perception areas. Particularly, activity in the left TPJ, a critical node in the speech perception/AVH network, appears to be disconnected from brain activity in areas involved in agency attribution, self-referential processing, and attention control (31).

According to Shergill (32), who also reported alterations in frontotemporal connections, insula activation occurred particularly when patients became aware of their hallucination and involved the Left Inferior Insula (LII), bilateral Superior Temporal Gyri (STG), bilateral Middle Temporal Gyri (MTG), Middle Frontal Gyrus (MFG), and Sensorimotor Cortex (SMC). Insula activation persisted after hallucinations and extended to the Orbitofrontal Cortex (OFC).

Frontal and temporal activations were reported in all resting-state functional MRI studies, with the involvement of additional systems such as ACC, insula, hippocampus, and TPJ. Connections between frontal and temporoparietal areas predominantly showed hypoactivity and were related to the FPN in relation to hyperactivity in systems such as the insula, hippocampus, and most components of the DMN, resulting in a “false attribution of inner speech” (27). This false attribution of inner speech, suggested as an explanation for AVH (29), is closely linked to dysfunction between language processing networks (BA, insula, SMA) and self-monitoring systems (DLPFC, VLPFC, IPC) (30). The alterations, however, were not only observed between frontotemporal connections and the mentioned adjacent systems but also at the interhemispheric level, between the left TPJ, anterior cingulate, and right BA. Furthermore, the importance of the emotional component of language was reported, both in its right hemisphere processing and in the decreased synchronization between TPJ activity and the amygdala, which may indicate reduced appreciation of verbal relevance during internal speech processing (31).

In conclusion, resting-state functional MRI studies suggest alterations between language processing networks that are closely linked to self-monitoring systems and involve important neural systems or networks such as ACC, insula, hippocampus, and amygdala. The disconnection, decoupling, and alterations in blood flow in these networks might not only affect language networks and systems, but also emotional processing systems that provide a broader perspective on AVH.

#### 3.2.2 Task-based studies

Task-based studies focusing on self-monitoring (33, 36) (**Table 3**) have shown that insight processes, divided into “self” and “other,” and self-monitoring are disrupted in patients with AVH due to alterations in networks involving bilateral thalamus, left lentiform nucleus (in the “self” condition), and alterations in the Posterior Cingulate Cortex (PCC), Inferior Parietal Lobule (IPL), insula, bilateral superior temporal cortices (STC), putamen, and right thalamus (in the “other” condition) (33). Furthermore, these studies revealed alterations in a network comprising bilateral Medial Geniculate Nucleus (MGN) of the thalamus, middle and superior temporal lobes (MTL and STL), inferior frontal cortex (IFC), insula, putamen, and Posterior Cingulate (PC). The increased response in MGN, along with MTL and IFC, coupled with reduced response in the Default Mode Network (DMN), primarily involving PC and Inferior Frontal Cortex (IFC), indicated faulty monitoring of internal speech processes in patients with AVH, with greater modulation in those presenting symptoms of blunted affect, emotional withdrawal, poor rapport, and passive social avoidance (36).

**Table 3:** Task functional MR studies.

Authors	Sample	Age (M/SD)	Gender (M/F)	Image Scope	Modality	Self-Monitoring Networks
Sapara, A. et al. (33)	42 (PRIG=13 POIG= 13 HC = 16)	(31.15/9.77) (37.85/7.43) (31.81/9.36)	(11/2) (9/4) (13/3)	ROI: ACC, DLPFC, IFG, TL, PCC, IPL, STG, Putamen.	Birchwood insight scale (BIS)	ACC, DLPFC, IFG, TL, PCC, IPL, STG, Putamen.
Matsumoto, K. et al. (34)	12 (AVH=6 HC=6)	(34.3/11.5) (34.0/7.9)	Not reported	Whole Brain	Story about Rosch’s ink stains	LSTG, LI
Vercammen, A. et al. (35)	22 (AVH = 22)	(36.18/12.31)	(11/11)	ROI: IFGop, IFGtri, insula, SMA, STS ACC, MTS, AG, SMG	Acentuación métrica	IFGop, IFGtri, insula, SMA, STS, ACC, CITM, AG, SMG
Kumari, V. et al. (36)	83 (AVH=63 HC=20)	(37.95/9.63) (33.95/10.37)	(74.6%/25.4%) HC (70%/30%)	Whole Brain	Verbal self-monitoring task	Thalamus (MGN), TL, IFG, MFG, PCC, STS
Simons, C. et al. (37)	27 (AVH=15 HC=12)	(34.7/8.7) (34.4/7.9)	Not reported	Whole Brain	Listening vs inner speech	LSTG, CG, IFG, ACC
Shergill, S. et al. (38)	16 (AVH=8 HC=8)	(31/9) (29/5)	Not reported	Whole Brain	Fast covert articulation vs slow covert articulation	BTC, PHC, RLC
Bentaleb, L. et al. (39)	2 (AVH=1 HC=1)	(36) (36)	(0/1) (0/1)	Whole Brain	External speech	LSTG, STS, PAC
Shergill, S. et al. (40)	14 (AVH=8 HC=6)	(32/10) (34/4)	Not reported	Whole Brain	verbal auditory imagery	PCC, BLN, RT, TCM, TCS, LNA

PRIG, Preserved Insight Group; POIG, Poor Insight Group; AVH, Auditory Verbal Hallucinations; NAVH, No Auditory Verbal Hallucinations; HC, Healthy Controls; M/SD, mean/standard deviation; M/F, male/female; ROI, Region of Interest; ACC, Anterior Cingulate Cortex; DLPFC, Dorsolateral Prefrontal Cortex; IFG, Inferior Frontal Gyrus; TL, Temporal Lobe; PCC, Posterior Cingulate Cortex; IPL, Inferior Parietal Lobe; STG, Superior Temporal Gyrus; LSTG, Left Superior Temporal Gyrus; LI, Left Insula IFGop, Inferior Frontal Gyrus Opercularis; IFGtri, Inferior Frontal Gyrus Triangularis; SMA, Supplementary Motor Area; STS, Superior Temporal Sulcus; MTS, Medial Temporal Sulcus; AG, Angular Gyrus; SMG, Supramarginal Gyrus; MGN, Medial Geniculate Nucleus; IFG, Inferior Frontal Gyrus; MFG, Medial Frontal Gyrus; TCS, Temporal Cortical Sulcus; CG, Cingulate Gyrus; BTC, Bilateral Temporal Cortex; PHC, Parahippocampal Cortex; PAC, Primary Auditory Cortex; RLC, Right Lateral Cerebellum; PCC, Posterior Cerebellar Cortex; BLN, Bilateral Lenticular Nucleus; RT= Right Thalamus; TCM, Temporal Cortical Medial; TCS, Temporal Cortical Superior; LNA, Left Nucleus Accumbens.

On the other hand, task-based studies involving some form of external speech processing (**Table 3**), such as listening vs. covert speech, slow covert articulation vs. fast covert articulation, and external speech, demonstrated that internal speech was associated with increased activation in the Inferior Frontal Cortex (IFC) and Anterior Cingulate Cortex (ACC) (37), and attenuated activation in the Bilateral Temporal Cortex (BTC), Right Parahippocampal Cortex (RPHC), and Right Lateral Cerebellum (RLC) (38). In contrast, the listening task was associated with activation in bilateral temporoparietal and occipital areas (37). In the absence of external speech, AVH patients exhibited activation in the Superior Temporal Gyrus (STG), Superior Temporal Pole (STP), and Primary Auditory Cortex (PAC) (39).

Finally, studies employing tasks involving internal processing and production (**Table 3**), such as the Rorschach inkblot task, metric accentuation, and verbal auditory imagery, reported the following: 1) reduced activation in STG and Left Insula (LI) during the generation of pauses and filled pauses between phrases in the inkblot task in AVH patients (34); 2) negative correlations between loudness and activation in bilateral ACC, bilateral Inferior Parietal Cortex (IPC), Inferior Triangular Frontal Cortex (ITFC), Inferior Opercular Frontal Cortex (IOFC), MTG, and LI, all involved in internal speech processing (35); and 3) association of internal speech with activation in IFC and insula, as well as attenuated activation in the Superior Temporal Cortex (STC), and verbal self-monitoring with temporal activation, which is relevant to AVH. Additional attenuated activations were observed in bilateral hippocampal complex, right thalamus, and left nucleus accumbens during verbal auditory imagery processes, which are also implicated in AVH (40).

In summary, task-based functional MRI studies focusing on internal speech processing have revealed alterations, particularly in temporoparietal areas, with frequent involvement of parahippocampal, cerebellar, thalamic, Posterior Cingulate, and Primary Auditory Cortex regions. These areas and networks, when disrupted, are associated with AVH.

### 3.3 Effective connectivity studies

#### 3.3.1 Resting-state studies

It has been reported that the Auditory Cortex (PAC), Left Dorsolateral Prefrontal Cortex (LDPFC), and Broca’s Area (BA) form a bidirectionally connected network in patients with AVH. This network exhibits reciprocal connectivity between the AC and the hippocampus, between the AC and the thalamus, between the AC and BA, and between the LDPFC and BA. Additionally, there is unilateral connectivity from the hippocampus to the LDPFC. This results in cortico-thalamo-auditory hyperconnectivity and cortico-hippocampal-auditory hypoconnectivity. The thalamo-AC hyperconnectivity leads to increased sensitivity of the AC to thalamic inputs in patients with AVH, which is associated with elevated AC activity in these patients. Disruption of this network, which is involved in language production (BA) and verbal self-monitoring (LDPFC), is associated with the default mode network (DMN), insula networks, striatal networks, and is implicated in AVH (42) (**Table 4**).

**Table 4:** Rest effective MRI studies.

Authors	Sample	Age (M/SD)	Gender (M/F)	Image Scope	Modality	Self-Monitoring Networks
Zhang, L. et al. (41)	73 (AVH=18 NAVH=18 HC=37)	(22.56/6.7) (22.67/3.85) (22.48/5.84)	(10/8) (9/9) (19/18)	ROI: LIFG, LMTG, LIPL	Resting state	LIFG, LMTG, LIPL
Li, B. et al. (42)	51 (AVH=17 NAVH=15 HC=19)	Not reported	Not reported	ROI: LDPFC, PAC, Hippocampus, Thalamus, BA	Resting state	LDPFC, PAC, Hippocampus, Thalamus, BA

AVH, Auditory Verbal Hallucinations; NAVH, No Auditory Verbal Hallucinations; HC, Healthy Controls M/SD, mean/standard deviation; M/F, male/female; ROI, Region of Interest; LIFG, Left Inferior Frontal Gyrus; LMTG, Left Middle Temporal Gyrus; LIPL, Left Inferior Parietal Lobe; LDPFC, Left Dorsolateral Prefrontal Cortex; PAC, Primary Auditory Cortex; BA, Broca’s Area.

Furthermore, decreased effective connectivity between the Left Inferior Frontal Gyrus (LIFG) and the Left Middle Temporal Gyrus (LMTG) has been reported, suggesting irregular patterns of causal interactions within the language network. There was also a decrease in effective connectivity from the Left Inferior Parietal Lobule (IPL) to the LMTG and from the LIFG to the LMTG, indicating a lack of effective inhibition from the Frontal Lobe (FL) on the internal verbal signal generated by the Temporal Lobe (TL), which in turn leads to an inability to integrate information related to internal speech. These deficits are associated with impaired self-monitoring and thus with AVH (41).

In conclusion, these studies generally report decreased effective connectivity in language networks, including subcortical and auditory areas.

#### 3.3.2 Task-based studies

According to the study performed by Mechelli et al. (44) (**Table 5**), participants were asked to indicate whether each word, manipulated in terms of their source and acoustic quality, was spoken in their own voice or in another person’s voice. Four conditions were measured: undistorted self-voice, distorted self-voice, undistorted other-voice, and distorted other-voice. The study found an intrinsic connection (the impact that one region exerts on another) between the Left Superior Temporal Cortex (LSTC) and the Anterior Cingulate Cortex (ACC). This connection, especially in the self-generated and other-generated speech conditions, was modulated by the source of speech. According to the study, this supports the hypothesis that dysfunction in the temporal-cingulate network could lead to false perceptions in schizophrenia.

**Table 5:** Task effective MRI studies.

Authors	Sample	Age (M/SD)	Gender (M/F)	Image Scope	Modality	Self-Monitoring Networks
Curcic, B. et al. (43)	53 (AVH=21 NAVH=14 HC=18)	(34/13) (30/5) (31/10)	(11/10) (13/1) (11/7)	ROI: LBA, RBA LWA, RWA	Metric accentuation	LBA, RBA LWA, RWA
Mechelli, A. et al. (44)	31 (AVH=11 NAVH=10 HC=10)	(35.33/6.63) (34.78/11.4) (28.50/4.37)	Not reported	ROI= LSTC, RSTC, LIFG, RFG, ACC	Manipulated Word Series	LSTC, RSTC, LIFG, RFG, ACC

AVH, Auditory Verbal Hallucinations; NAVH, No Auditory Verbal Hallucinations; HC, Healthy Controls; M/SD, mean/standard deviation; M/F, male/female; ROI, Region of Interest; LBA, Left Broca’s Area; RBA, Right Broca’s Area; LWA, Left Wernicke’s Area; RWA, Right Wernicke’s Area; LSTC, Left Superior Temporal Cortex; RSTC, Right Superior Temporal Cortex; LIFG, Left Inferior Frontal Gyrus; RIFG, Right Inferior Frontal Gyrus; ACC, Anterior Cingulate Cortex.

In a task involving metric accentuation, which aimed to measure internal speech processing, reduced connectivity was found between bilateral Auditory Wordform Area (AW) and Broca’s Area (BA), as well as from the left BA to the right BA. The reduction of information flow from AW to BA may lead to a loss of feedback (self-monitoring) and an increase in top-down efforts that are less constrained by perceptual information from BA. This has been theorized as a possible cause of AVH. The reduction of information flow from the left BA to the right BA appears to be associated with the emotional content of hallucinations, as the Right Inferior Frontal Gyrus (RIFG) has been implicated in emotional aspects of speech and monitoring processes (43).

In conclusion, task-based effective connectivity studies, in line with resting-state studies, suggest altered information flow in both the frontotemporal network and networks involving the interaction of these regions with the ACC. These alterations not only have effects on self-monitoring and internal speech processes but also on the emotional aspects conveyed by language.

## 4 Discussion

This research systematically reviewed the published studies to date on fMRI that examined the neuropsychological dimensions related to verbal self-monitoring impairments in schizophrenic language. The significant methodological heterogeneity among the articles precluded a statistical analysis. Despite this limitation, several important conclusions can be drawn from this review, even though only a few articles directly addressed neuropsychological dimensions other than language.

Without exception, all studies indicated some form of language network alteration in patients with schizophrenia, supporting the hypothesis that language and its impairments are fundamental elements of the disorder (3, 4, 18, 2). As noted in some studies (12, 45), inner speech is a crucial component of verbal self-monitoring processes, and its disturbances, which can lead to misattribution or externalization, appear to provide a consistent explanation for auditory verbal hallucinations (AVHs), as demonstrated particularly by functional MRI studies. As mentioned in the introduction, verbal self-monitoring is involved in the generative process of language, which encompasses the transition from sense to meaning and determines language processing (encoding-decoding). Disruptions in this process due to brain lesions result in various forms of aphasia (14). However, when impairments are due to aberrations in connectivity, as in schizophrenia, symptomatic manifestations are expected to acquire a distinct character.

While it was found that verbal self-monitoring processes are closely related to frontal areas (29, 30), their complexity suggests that, rather than frontal lobe dysfunctions (10), altered verbal self-monitoring in schizophrenia involves frontotemporal parietal networks, with significant involvement of systems such as the putamen (33, 36), hippocampus (27, 38, 42), thalamus (36, 42), amygdala (31), and notably, the Cingulate Cortex (24, 28, 31, 33, 37, 44, 35), insula (27, 28, 32–36), the default mode network (DMN), and the frontoparietal network (FPN) (27). Therefore, the specificity of language impairments in schizophrenia appears to be a consequence of the multiplicity of affected areas and networks, both at the structural, functional, and effective levels, resulting in general language processing impairments and other neuropsychological dimensions. This is in contrast to aphasia, where more specific neural areas and networks are affected, allowing for the dissociation of its symptoms from other neuropsychological functions (46). Thus, these findings are somewhat consistent with Frith’s (19) classic model of verbal self-monitoring, thereby expanding the perspective on frontal dysfunctions in the neuropsychology of schizophrenic language.

Based on these findings, it is suggestive to propose understanding schizophrenia as an impairment that affects various levels of the language generative process, with the presence of verbal self-monitoring within these levels. These alterations are related to abnormal constructions of meaning during comprehension, perception, action, and production of language, giving rise to delusions (comprehension), hallucinations (perception), disorganized behavior (action), and disorganized speech or thought disorder (production) (47). Verbal self-monitoring also influences other neuropsychological dimensions, particularly perception (33, 39) - which integrates the theory of auditory perception and inner speech of AVHs - and emotion (31, 43). This suggests the need for studies that place language as a fundamental focus in schizophrenia and, with a solid theory of language processing, determine its specific relationship with schizophrenia.

In the relationship between language, other neuropsychological dimensions, and schizophrenia, studies on effective connectivity are of particular interest. These studies allow for the estimation and inference of the influence that one neuronal system or network exerts on another, either directly or indirectly, through Dynamic Causal Models (DCM) (48). In other words, structural connectivity enables spatial correlations, functional connectivity enables temporal correlations, and effective connectivity enables dynamic correlations between functional systems or networks. Consequently, the influence of language networks on other neuropsychological functions, both in healthy populations and in populations with neuropsychiatric disorders such as schizophrenia, could be better estimated using these types of measures. In relation to the findings, it is not only important to know that there are alterations in frontotemporal networks and other mentioned neuronal groups, but it is also relevant to understand, for example, how the information flow from the anterior to the posterior language regions is altered. This knowledge can help establish stimulation processes that emphasize tasks aimed at compensating for these informational alterations. Therefore, the knowledge or findings of effective connectivity can be a promising avenue for the study of neuropsychological rehabilitation in schizophrenia.

According to Andreasen (49), the neuropsychological approach to schizophrenic language should propose three fundamental objectives: clinical description of the phenomena, functional or cognitive mapping of the presented deficits, and neuroanatomical mapping based on functional deficits. The findings allow for direct neuroanatomical mapping based on the networks and their (hypo-hyper) structural, functional, and effective connectivity alterations. They also enable functional or cognitive mapping based on the affected neuropsychological dimensions beyond language. However, the clinical description, to the extent that it depends on neuroanatomical and functional networks and the processes that could compensate for their alterations, is a posteriori construction that should provide practical elements for neuropsychological intervention (52, 53). Currently, these constructions can take at least two clinical-practical paths: the development of innovative neuroimaging techniques and representations that use these data as measures for the diagnosis and treatment of schizophrenia, such as the SpeechGraphs project of the “Instituto do Cérebro” in Brazil, which uses Graph Theory and develops speech graphs software for diagnostic processes and suggestions for neurolinguistic interventions in schizophrenia (50, 51); and the development of neuropsychological tests for schizophrenia that emphasize language tasks, particularly tasks involving the activation (hypo-hyper, as the case may be) of inner speech - due to its relationship with the positive symptomatology of schizophrenia - such as verbal auditory imagery.

One final point of discussion and limitation is the absence of theoretical justification regarding the selection of age in the sampled articles. Although some of them studied the early appearances of AVHs, which could explain the inclusion of patients aged between 18 and 25 years, this was not explicitly stated in all articles. Age in schizophrenia can be relevant, especially when studied in relation to language, as symptomatology can vary depending on the patient’s developmental stage and the progression of the disease. Another limitation, as mentioned before, was the heterogeneity of the data, which did not allow for quantitative analysis of the results.

## 5 Conclusion

Despite the high methodological heterogeneity and the variability in reported networks among the reviewed articles, there is a general consensus suggesting that language and its verbal self-monitoring mechanisms are not secondary in schizophrenia but rather fundamental to the disorder. These mechanisms are also related to alterations in other neuropsychological dimensions, particularly emotional and cognitive dimensions such as perception. The networks that showed the most commonalities form a complex system involving frontotemporal networks and regions such as the insula, amygdala, putamen, and cingulate cortex, which are associated with the default mode network (DMN) and frontotemporal networks (FTN). These findings provide valuable insights for the development of techniques and tests that enable the diagnosis and treatment of schizophrenia from a neuropsychological perspective. Language tasks involving the processing of inner speech, such as verbal auditory imagery tasks, may be particularly relevant in this regard.

## Data availability statement

The original contributions presented in the study are included in the article/supplementary material. Further inquiries can be directed to the corresponding author.

## Author contributions

JG: Conceptualization, Data curation, Formal analysis, Funding acquisition, Investigation, Methodology, Project administration, Resources, Software, Supervision, Validation, Visualization, Writing – original draft, Writing – review & editing.
